# Long-term spring through fall capture data of *Eptesicus fuscus* in the eastern USA before and after white-nose syndrome

**DOI:** 10.1016/j.dib.2023.109353

**Published:** 2023-06-28

**Authors:** Molly C. Simonis, Lynn K. Hartzler, Joshua Campbell, Timothy C. Carter, Lisa Noelle Cooper, Katelin Cross, Katherine Etchison, Traci Hemberger, R. Andrew King, Richard J. Reynolds, Yasmeen Samar, Michael R. Scafini, Sarah Stankavich, Gregory G. Turner, Megan A. Rúa

**Affiliations:** a Wright State University, Environmental Sciences PhD Program, Dayton, OH, United States; b University of Oklahoma, Department of Biology, Norman, OK, United States; c Wright State University, Department of Biological Sciences, Dayton, OH, United States; d Tennessee Wildlife Resources Agency, Nashville, TN, United States; e Ball State University, Department of Biology, Muncie, IN, United States; f North Eastern Ohio Medical School, Musculoskeletal Research Focus Area, Department of Anatomy and Neurobiology, Rootstown, OH, United States; g Mississippi Department of Wildlife, Fisheries, and Parks, Jackson, MS, United States; h Mississippi Museum of Natural Science, Jackson, MS, United States; i North Carolina Wildlife Resources Commission, Raleigh, NC, United States; j Kentucky Department of Fish and Wildlife Resources, Frankfort, KY, United States; k US Fish and Wildlife Service, Indiana Field Office, Bloomington, IN, United States; l Virginia Department of Wildlife Resources, Verona, VA, United States; m University of Pennsylvania, School of Veterinary Medicine, Philadelphia, PA, United States; n Pennsylvania Game Commission, Harrisburg, PA, United States; o Bat Conservation International, Austin, TX, United States; p Ohio Division of Wildlife, Columbus, OH, United States

**Keywords:** Bats, Big brown bat, Capture records, Emerging infectious disease, Mammals, Mist net, *Pseudogymnoascus destructans*

## Abstract

Emerging infectious diseases threaten wildlife populations. Without well monitored wildlife systems, it is challenging to determine accurate population and ecosystem losses following disease emergence. North American temperate bats present a unique opportunity for studying the broad impacts of wildlife disease emergence, as their federal monitoring programs were prioritized in the USA throughout the 20^th^ century and they are currently threatened by the invasive fungal pathogen, *Pseudogymnoascus destructans* (*Pd*), which causes white-nose syndrome. Here we provide a long-term dataset for capture records of *Eptesicus fuscus* (big brown bat) across the eastern USA, spanning 16 years before and 14 years after *Pd* invasion into North America. These data represent 30,496 *E. fuscus* captures across 3,567 unique sites. We encourage the use of this dataset for quantifying impacts of wildlife disease and other threats to wildlife (e.g., climate change) with the incorporation of other available data. We welcome additional data contributions for *E. fuscus* captures across North and Central America as well as the inclusion of other variables into the dataset that contribute to the quantification of wildlife health.

**Specifications Table**

**Table:** 

Subject	Environmental Science: Ecology
Specific subject area	Capture data of *Eptesicus fuscus* in spring through fall months from 1990-2020 paired with spatiotemporal spread of *Pseudogymnoascus destructans*.
Type of data	Table
How the data were acquired	Capture data were acquired from government wildlife agencies and bat researchers from Georgia, Illinois, Indiana, Kentucky, Mississippi, New York, North Carolina, Ohio, Pennsylvania, Tennessee, and Virginia. Raw data for *Eptesicus fuscus* captures were supplied and data were collated. We also paired capture data with data for the spatiotemporal spread of *Pseudogymnoascus destructans*, which was acquired from the US Geological Survey map application at whitenosesyndrome.org/where-is-wns.
Data format	Filtered, Analyzed, Secondary
Description of data collection	Historical mist net capture data were opportunistically collected from government wildlife agencies and bat researchers. We collected data for mass, forearm length, age, sex, reproductive status, capture date and location. We cleaned data by removing entries with missing or unclear values, and masked sensitive location data. Data for the first year of pathogen introduction within each state (from the whitenosesyndrome.org/where-is-wns US Geological Survey map application) was paired by the year of each capture.
Data source location	Data were obtained from the following organizations and government agencies by contacting affiliated authors or acknowledgements via email • Ball State University, Department of Biology, Muncie, IN, USA • Georgia Department of Natural Resources Wildlife Resources Division, Social Circle, GA, USA • Kentucky Department of Fish and Wildlife Resources, Frankfort, KY, USA • Mississippi Department of Wildlife, Fisheries, and Parks, Jackson, MS, USA • Mississippi Museum of Natural Science, Jackson, MS, USA • New York State Department of Environmental Conservation, Albany, NY, USA • North Carolina Wildlife Resources Commission, Raleigh, NC, USA • North Eastern Ohio Medical School, Department of Anatomy and Neurobiology, Rootstown, OH, USA • Ohio Department of Natural Resources Division of Wildlife,
	Columbus, OH, USA • Pennsylvania Game Commission, Harrisburg, PA, USA • Tennessee Wildlife Resources Agency, Nashville, TN, USA • US Fish and Wildlife Service, Indiana Field Office, Bloomington, IN, USA • US Fish and Wildlife Service North Carolina Field Office, Asheville, NC, USA • Virginia Department of Wildlife Resources, Verona, VA, USA
Data accessibility	Repository Name: Dryad Digital Repository Data Identification Number: 10.5061/dryad.ngf1vhhvv Direct Link to Data: https://doi.org/10.5061/dryad.ngf1vhhvv[1]
	Repository Name: GitHub; Zenodo Data Identification Number: 10.5281/zenodo.7799825 Direct Link to Code: https://zenodo.org/record/7799825#.ZCyLjHbMLIU[2]
Related research article	M.C. Simonis, L.K. Hartzler, G.G. Turner, M.R. Scafini, M.A. Rúa, Long-term exposure to an invasive fungal pathogen decreases *Eptesicus fuscus* body mass with increasing latitude, Ecosphere (2023), 14(2), e4426. https://doi.org/10.1002/ecs2.4426[3]

## Value of the Data

• Understanding how wildlife populations change following an impact is critical as human-induced disturbances can influence impact frequency and magnitude, as suggested for the growing number of emerging infectious diseases in wildlife [4,5].
• Emerging infectious diseases threaten wildlife to, or near, extinction, and without sufficient data prior to disease emergence, management strategies to conserve wildlife species may be unsuccessful.
• Without data before disease emergence, calculating true losses to ecosystem services and function is extremely challenging. Thus, well monitored wildlife systems can help clarify how populations change following impacts from emerging infectious disease.
• Here, we pair long-term capture records of *Eptesicus fuscus* (big brown bats) with spatiotemporal spread of *Pseudogymnoascus destructans*, the invasive fungal pathogen causing white-nose syndrome [6,7]. These data consist of spring through fall capture records spanning 16 years before and 14 years after the first detection of *Pd* in New York, USA, in 2006, where it was introduced from Eurasia [6,[8], [9], [10].
• This *E. fuscus* capture dataset provides spatial and temporal data for both wildlife host and pathogen spread, but data are not limited to usage for only disease impact research. These data can be paired with other datasets presenting other conservation threats to bats with a county-level spatial resolution (e.g. climate change impacts, agricultural and urbanization intensification impacts, insect decline impacts, etc.).
• We welcome additional contributions to this dataset for past and future *E. fuscus* capture records across their species range throughout North and Central America. We also welcome the inclusion of additional variables into the dataset such as *Pd* intensity and/or any metric representative of bat health upon capture (e.g., heavy metal concentrations, differential white blood cell counts, cortisol concentration, other present pathogens, etc.).

## 1 Objective

Our objective is to provide accessible, long-term data for a well monitored wildlife system impacted by an emerging infectious disease. Bat host species that are less susceptible to *Pd* infection (relative to highly susceptible species) persist despite annual winter infections and thus, can inform how long-term pathogen exposure impacts persisting host populations. *Eptesicus fuscus* (big brown bat) are classified as less susceptible to *Pd* infections and their populations persist despite annual winter infections [11,12]. *E. fuscus* in the eastern USA are relatively well monitored in spring through fall months (compared to other wildlife systems) because they are a common by-catch during federally endangered *Myotis sodalis* (Indiana bat) summer capture surveys, setting up the opportunity for a long-term dataset representing before and after the impact of an emerging infectious disease. Here, we provide a description of a dataset representing 30 years of *E. fuscus* spring through fall capture data across 11 eastern USA states paired with *Pd* introduction and invasion timing [1]. This dataset was previously used to quantify morphometric trait shifts [3] and capture rate changes across space over *Pd* invasion time (Simonis *et al*.; in review). We encourage future contributions to this dataset and its use for impact studies requiring long-term wildlife records. Future contributions to this dataset can be made by contacting the corresponding author of this manuscript, and newly integrated data will be made available through Dryad Digital Repository [1].

## 2 Data Description

**Fig. 1:**
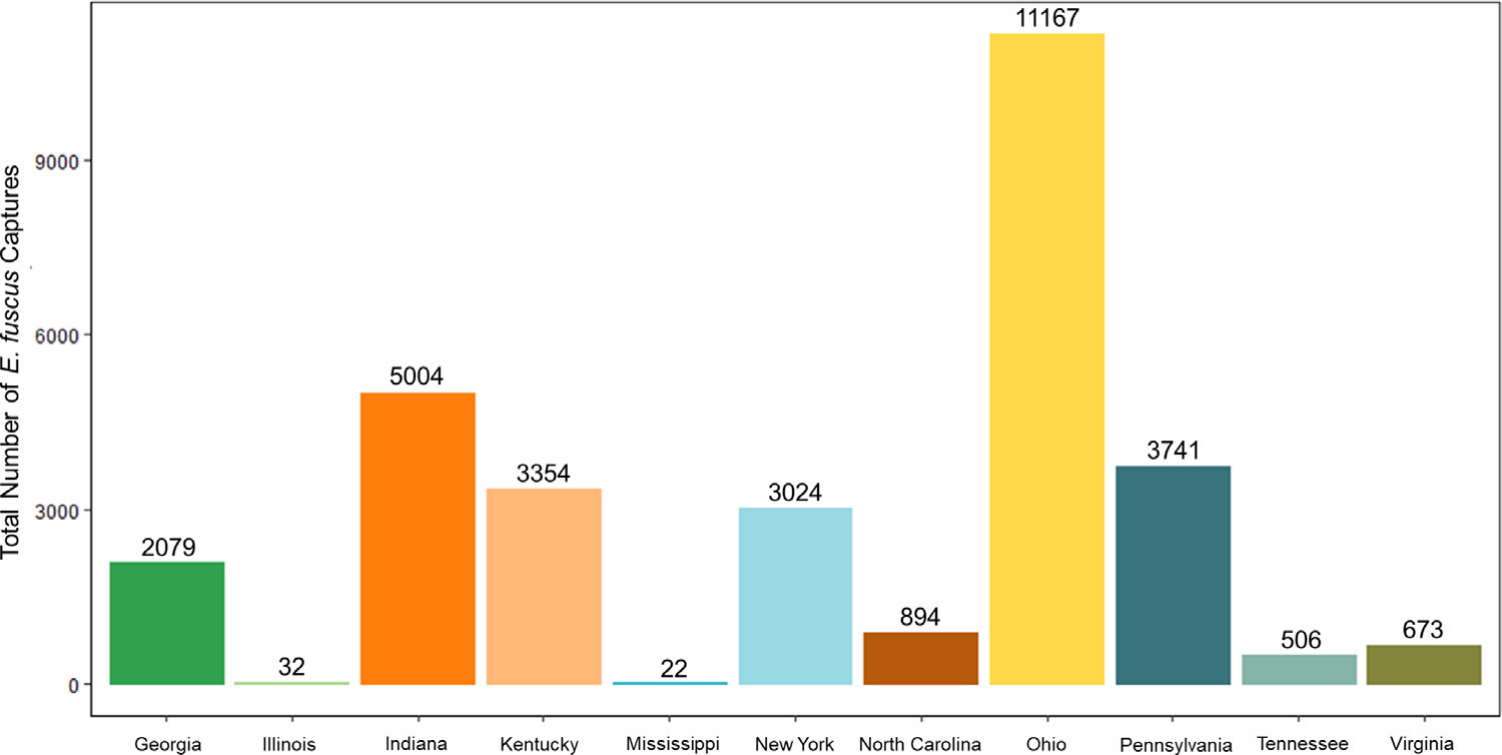
The number of *Eptesicus fuscus* capture records varied by the USA state they were collected from within the dataset. Numbers above each bar are total *E. fuscus* captures for each state within the dataset.

**Fig. 2:**
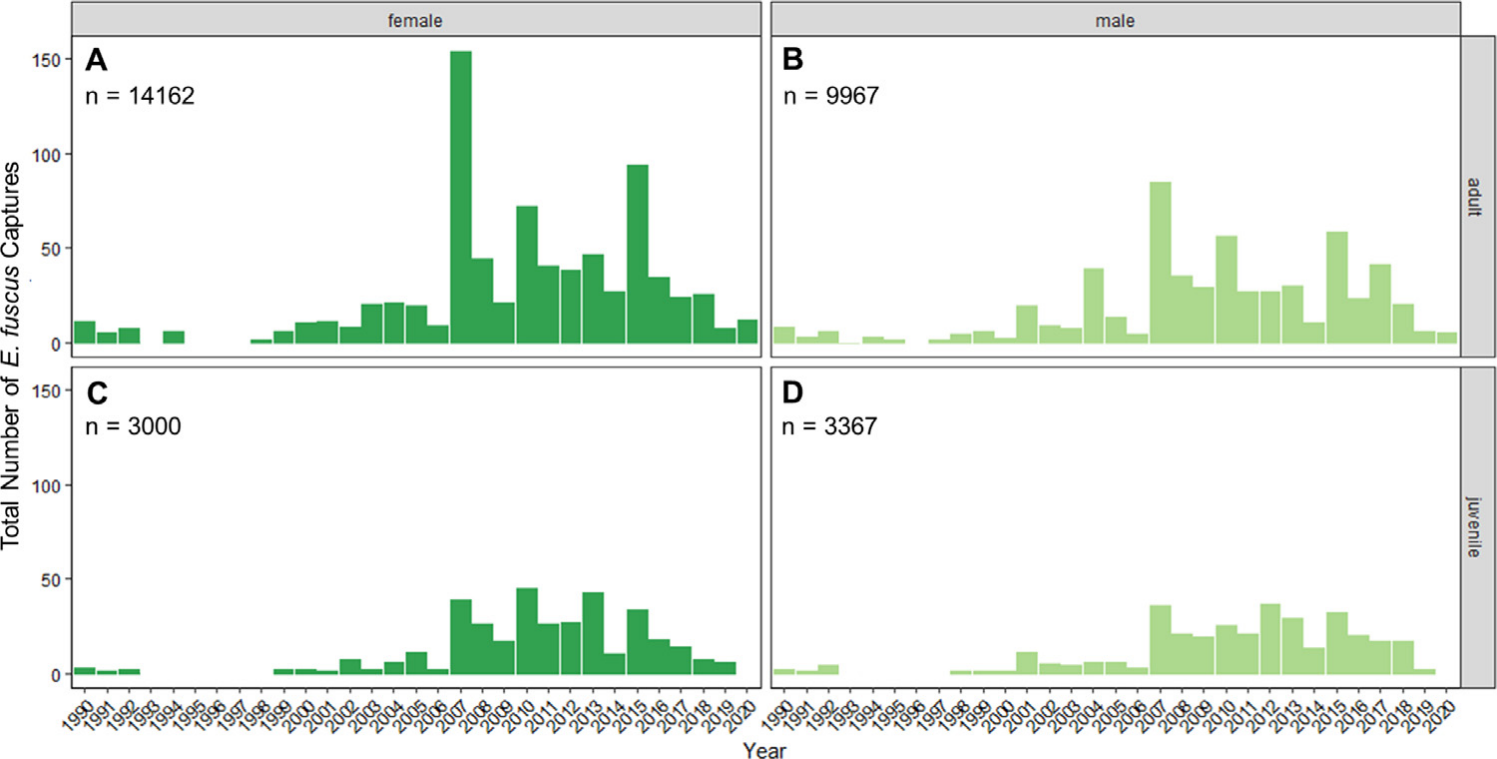
The number of *Eptesicus fuscus* capture records varied by year for A) adult females, B) adult males, C) juvenile females and D) juvenile males. Note that there were no *E. fuscus* capture data collected in 1993 and 1996 for any sex or age category. Numbers within each plot are total *E. fuscus* captures across all years for each sex and age category.

This dataset [1] consists of 30,496 *E. fuscus* captured across 11 eastern USA states (Fig. 1) within the months of May through October and between the years of 1990 to 2020 (Fig. 2). The total amount of capture records in this dataset within each state are as follows: Georgia, 2,079; Illinois, 32; Indiana, 5,004; Kentucky, 3,354; Mississippi, 22; New York, 3,024; North Carolina, 894; Ohio, 11,167; Pennsylvania, 3,741; Tennessee, 506; and Virginia, 673 (Fig. 2).

**Fig. 3:**
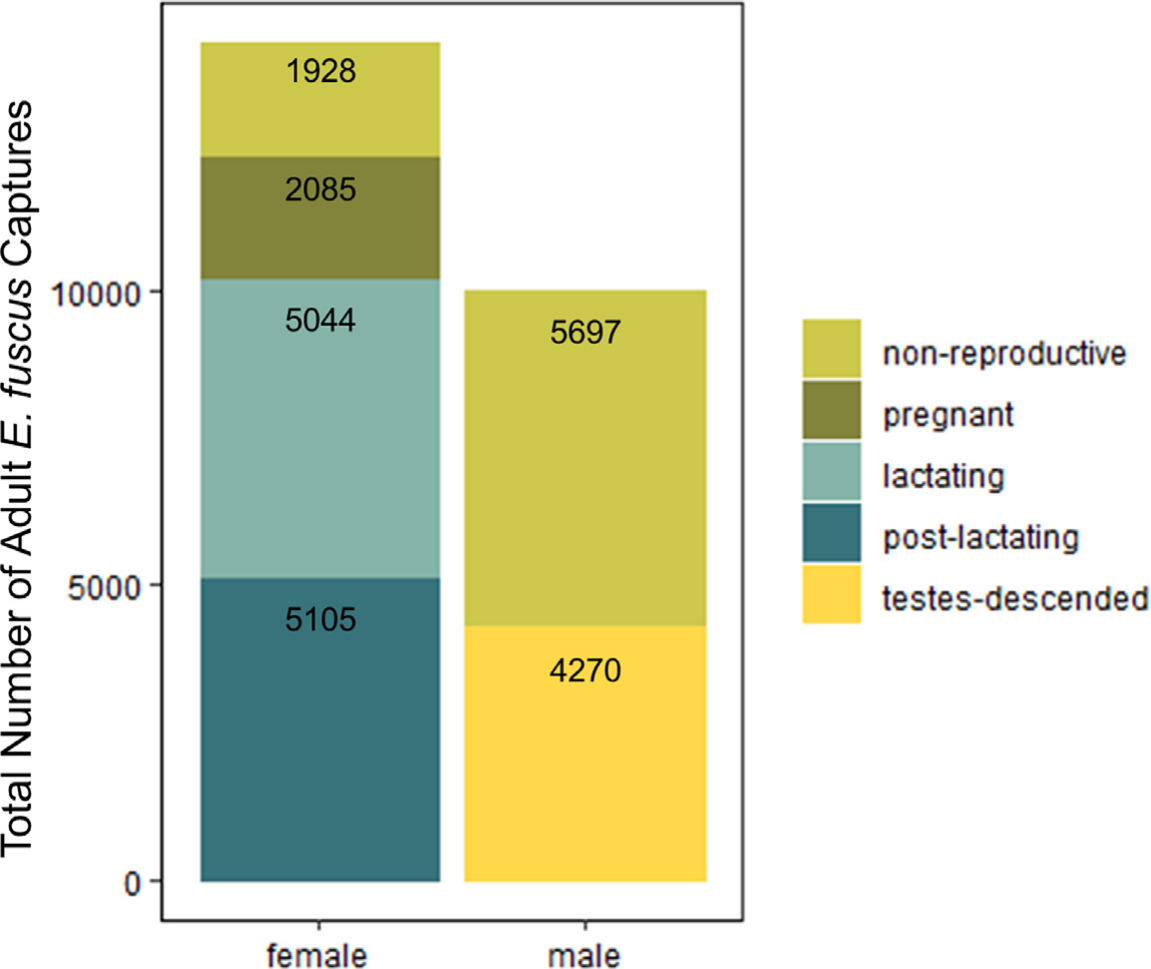
The quantity of adult *Eptesicus fuscus* capture records varied by sex and their reproductive status. Numbers within each stack are total *E. fuscus* captures within each reproductive status for females or males. Note that while reproductive adult males are represented in this dataset (testes-descended), they are likely not actively reproductive throughout all capture months represented in this dataset (March through October). Color represents reproductive status.

**Fig. 4:**
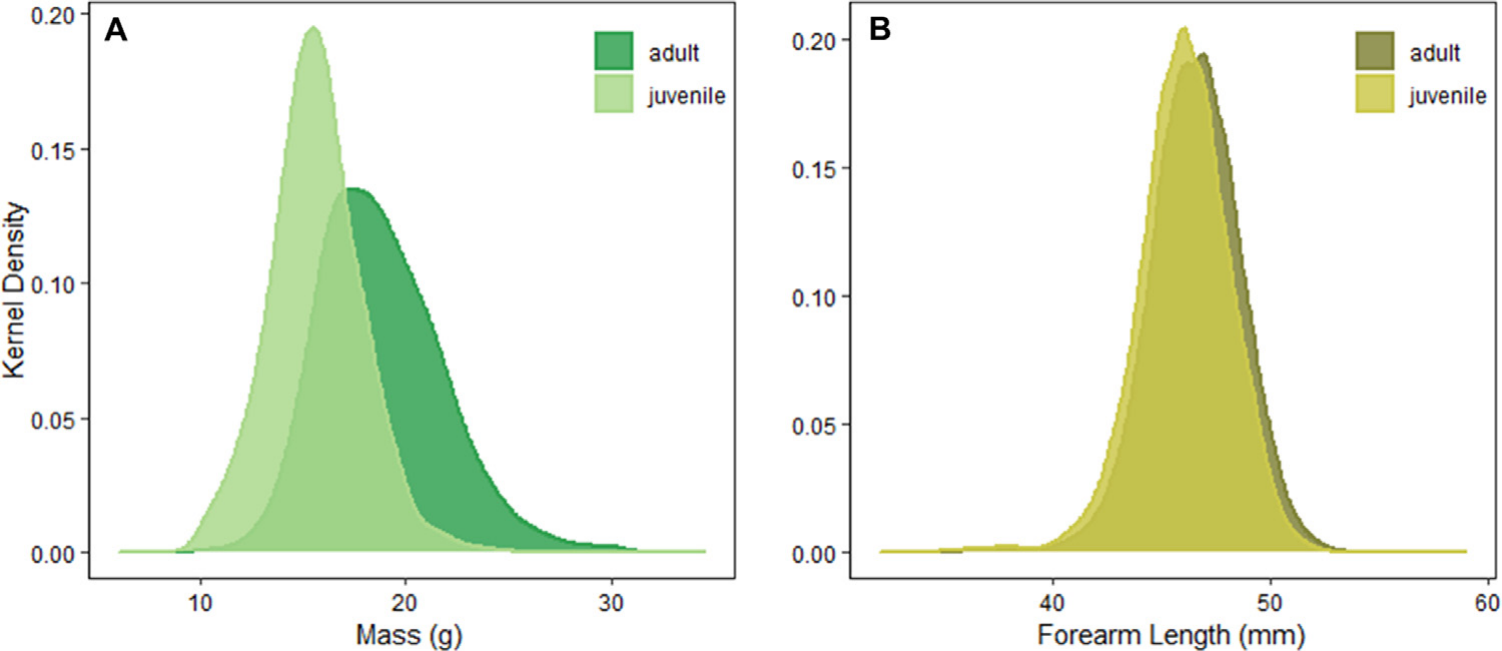
Distributions of A) mass and B) forearm length for all adult and juvenile *Eptesicus fuscus* within the dataset. Colors represent ages of bats for both A) mass and B) forearm length. Density plots were set on a bandwidth of 0.5 for both mass (A) and forearm length (B).

Overall, this dataset [1] includes *E. fuscus* capture records representing 14,162 adult females (Fig. 2A), 9,967 adult males (Fig. 2B), 3,000 juvenile females (Fig. 2C) and 3,367 juvenile males (Fig. 2D). The number of *E. fuscus* capture records within these age and sex demographics vary across the year of capture but, in general, increase over time (Fig. 2). Within adult-aged *E. fuscus*, the amount of capture records within the dataset also varies by their reproductive status at the time of capture. For adult females, the dataset consists of 1,928 non-reproductive bats, 2,085 pregnant bats, 5,044 lactating bats and 5,105 post-lactating bats (Fig. 3). For adult males, 5,697 bats captured are non-reproductive and 4,270 bats were captured with descended testes (Fig. 3). Finally, mass and forearm length varied by *E. fuscus* age and sex at time of capture with juveniles generally weighing less than adults (Fig. 4A) and no distinguishable differences in forearm lengths by age across the dataset (Fig. 4B).

**Fig. 5:**
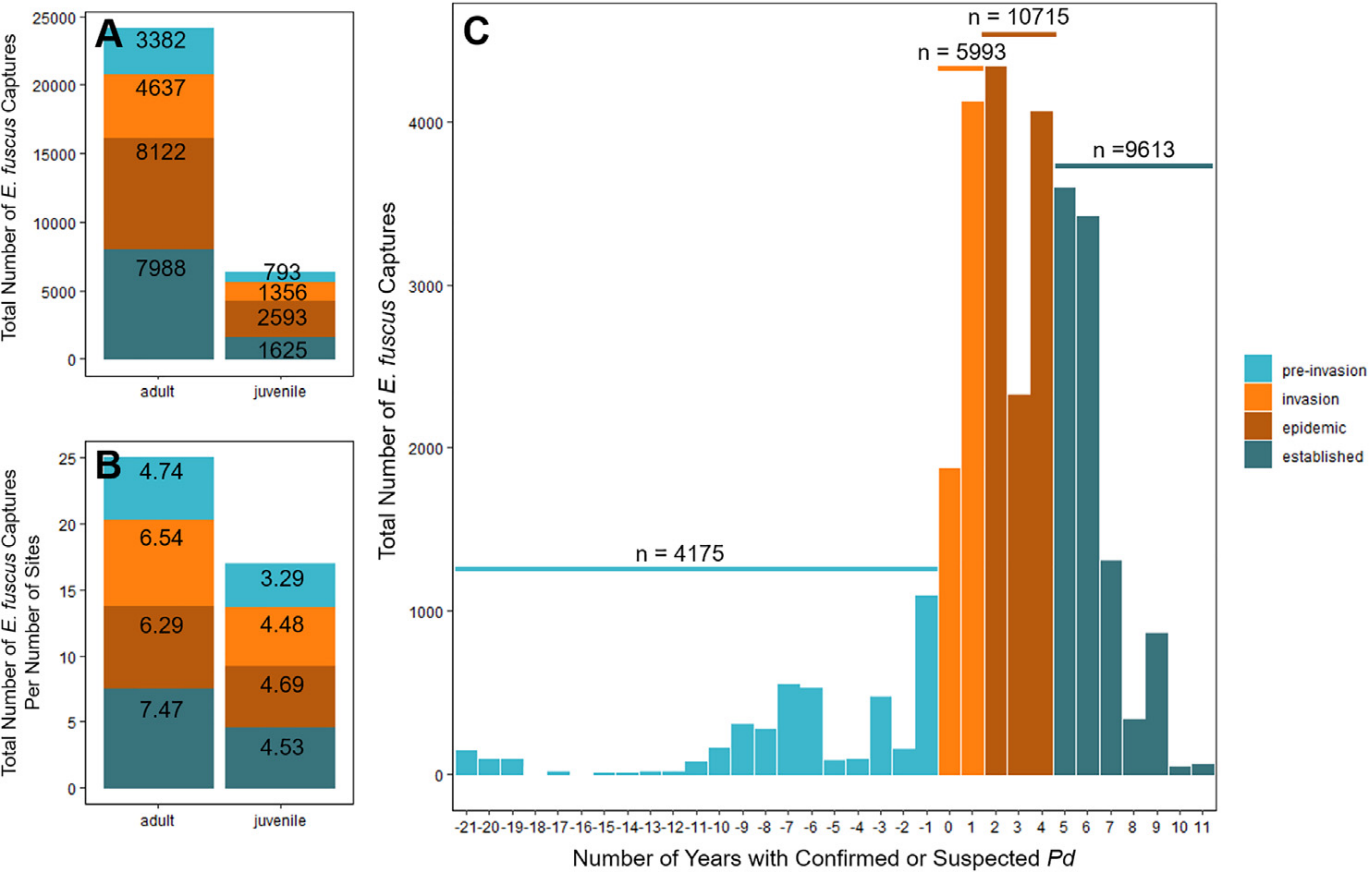
The number of *Eptesicus fuscus* capture records in this dataset vary by pathogen invasion time-step. A) The number of adult or juvenile captures are dependent on *Pseudogymnoascus destructans* (*Pd*) invasion time-steps. Numbers within each stack are total *E. fuscus* captures within each *Pd* invasion time-step. B) The number of adult or juvenile captures per site remained relatively stable across *Pd* invasion time-steps. Numbers are the total number of *E. fuscus* captures divided by the total number of capture sites within each *Pd* invasion time-step (effort). C) The number of all *E. fuscus* captures vary across the number of years since confirmed or suspected *Pd*, where ‘0’ is the year *Pd* was first detected and/or suspected in each state of capture. Note that there were no *E. fuscus* capture data collected at -16 and -18 years with confirmed or suscpected *Pd*. Numbers are total *E. fuscus* captures across each *Pd* invasion time-step spanning their respective time periods (horizontal lines). Colors represent *Pd* invasion time-steps.

The amount of *E. fuscus* capture records also generally increased across *Pd* invasion time-steps (Fig. 5A); however, the number of capture sites also increased causing the number of bats captured per site within each time-step (effort) to remain relatively stable for adult and juvenile *E. fuscus* (Fig. 5B). *E. fuscus* captures also generally increased over the years since confirmed or suspected *Pd* invasion within each time-step (Fig. 5C). Pre-invasion years consisted of 4,175 bat captures (3,382 adults and 793 juveniles), invasion years had 5,993 bat captures (4,637 adults and 1,356 juveniles), epidemic years had 10,715 bat captures (8,122 adults and 2,593 juveniles) and established years had 9,613 bat captures (7,988 adults and 1,625 juveniles; Fig. 5A & 5C).

*E. fuscus* capture data (“SIMONIS_et_al_BigBrownBatData_Dryad.csv”) and variable descriptions (“SIMONIS_et_al_BigBrownBat_README_20220725.txt”) can be accessed via Dryad Digital Repository [1]. Code for descriptive data figures (Fig. 1–5) presented in this manuscript (“DataDescriptor_20221026.Rmd”) with its associated description (“README.md”) can be accessed via MCS’ Github page (https://github.com/simonimc/Eptesicus_fuscus_big_brown_bat_data_descriptor_figures) and/or through Zenodo [2].

## 3 Experimental Design, Materials and Methods

**Fig. 6:**
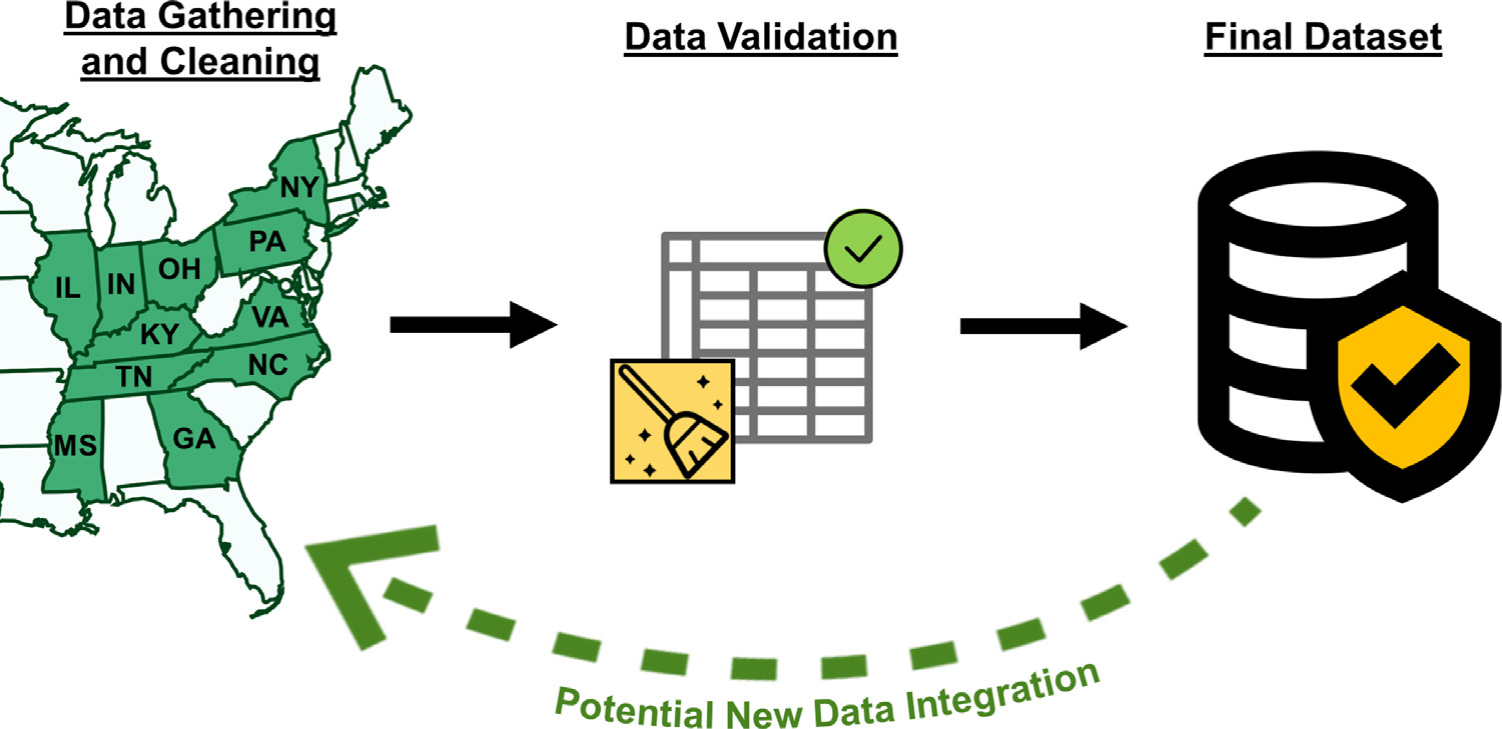
We gathered and collated *Eptesicus fuscus* capture records and *Pseudogymnoascus desctructans* introduction timing from 11 USA states and validated individual entries to create a complete final dataset. New data integration can be incorporated by contacting the corresponding author.

We gathered and collated historical *E. fuscus* mist net capture data from 11 USA states between July 2018 through May 2021 (Fig. 6). Data were opportunistically collected from federal wildlife agencies, state wildlife and natural resource agencies, and individual wildlife researchers. Government and academic representatives were contacted via email, and those with available data (coauthored above or acknowledged below) provided state mist net capture data through email communication. Variables of interest within the gathered *E. fuscus* data included: date of capture (month, day and year), USA state of capture (Georgia, Illinois, Indiana, Kentucky, Mississippi, New York, North Carolina, Ohio, Pennsylvania, Tennessee, or Virginia), site name of capture, county of capture, demographic state of individual bat (adult or juvenile), sex of individual bat (male or female), reproductive status of individual bat (female: non-reproductive, pregnant, lactating, post-lactating; male: testes-descended), mass of individual bat (g) and forearm length of individual bat (mm).

Once collected and collated, the raw dataset consisted of 40,689 individual *E. fuscus* captures. If the date of capture, demographic state, sex, reproductive status, mass or forearm length was missing from individual *E. fuscus* capture entries (left blank or entered as varying versions of “unknown” or “NA”), the entry was removed from the raw dataset. We also removed entries with unclear reproductive status entries. For example, if an adult female was marked as “reproductive” without indication of reproductive stage (*i.e.,* pregnant, lactating or post-lactating), the entry was removed.

For “age” (demographic state), “sex” and “repstat” (reproductive status) variables, we grouped varying versions of entries into distinct levels within each variable using filtering and find and replace tools in Microsoft Excel. Within the “age” variable, entries such as “A”, “AD” and “Adult” were labeled as “adult” and entries like “J”, “Juvi” and “JUV” were labeled as “juvenile”. Within the “sex” variable, reported values such as “F”, “Female” and “fem” were labeled as “female” and “M” and “Male” as “male”. The “repstat” variable underwent a similar process where entries such as “N”, “NR”, “up” and “non” became “non-reproductive”; entries such as “P”, “PR” and “PG” became “pregnant”; entries with versions of “L”, “lact” and “LA” became “lactating”; entries with entries like “PL”, “post lac”, “post” became “post-lactating; and labels such as “TD”, “scrotal”, “S” and “down” became “testes-descended”. Finally, in instances where entries for “age” and “sex” and/or “repstat” were placed under the wrong variable, we manually corrected the entry. For example, if a bat had an “age” of “post-lactating”, a “sex” of “adult” and a “repstat” of “female”, we correct the entry so the bat had an “age” of “adult”, a “sex” of “female” and a “repstat” of “post-lactating”.

We filtered the dataset to only keep *E. fuscus* captures that occurred between the months of March through October which corresponds to records for spring through fall. We only kept spring through fall records because 1) bats in winter months are typically not active when they are captured and, thus, different capture methods are used and 2) winter records were not explicitly requested. If neither the county of capture or latitude and longitude of capture were not provided, those entries were also removed except in cases where the site name or site description could be found to the county level online. When county of capture was provided, we used the reported county name. If latitude and longitude of capture were provided without a county of capture, we determined the county of capture by linking the spatial point provided to its respective county using the *sp, maps*, and *maptools* package in the statistical environment R [13], [14], [15], [16], [17]. To do so, we adapted publicly available R code for a function created to match a spatial point to a state but instead, matched to a county (see https://github.com/simonimc).

If site of capture was not provided, we allowed a site description to take its place if available. For example, for a general location described as “Ash Creek @ Hwy”, the site name became “Ash Creek @ Hwy”. If neither a site nor a description was provided, we then created a site name using the naming pattern “No Site Name <county of capture>”. Site names were then manually cleaned in OpenRefine version 3.5 (available at https://openrefine.org/) to ensure variations in site names within each county and state were labeled as a single site. For example, if site names were labeled by capture nets (e.g., “Site 1 Net A”, “Site 1 Net B”), we pooled nets under the single site name (e.g., “Site 1 Net A” and “Site 1 Net B” both become “Site 1”). Another example would be if multiple sites names were listed under the same general location (e.g., “Mammoth Cave National Park Site 1”, “Mammoth Cave National Park Site 2”), we compiled those sites within the general location (e.g., “Mammoth Cave National Park Site 1” and “Mammoth Cave National Park Site 2” both became “Mammoth Cave National Park”). Finally, if spelling errors occurred across a single site name, we corrected the site name to the correct spelling (e.g. “Mamoth Cav National Park” became “Mammoth Cave National Park”).

Due to the sensitivity of some of these data for disclosing locations of federally listed endangered or threatened bat species, we masked location and site data. Masking location and site data across the entire dataset also upheld data agreement terms and conditions contracted with Indiana, Kentucky and Tennessee. Once the county of each bat captured was identified as described above, we determined a county centroid point for each individual capture using the *housingData* package [18]. Thus, we created additional variables within the dataset for the latitude and longitude of those county centroid points, setting the spatial resolution of bat captures at the county level. To further ensure sensitive geographic data were not exposed, we also masked site names within the dataset. We masked sites by labeling each site with a unique identifier within the state. For example, “Site 1” in Georgia became “GA_01”. Following initial data collation and cleaning, 30,496 individual bat captures across 3,567 unique sites remained in the dataset (Fig. 6).

In addition to *E. fuscus* capture records, we added variables for geographic spread over time of the invasive fungal pathogen *Pseudogymnoascus destructans* (*Pd*), which causes white-nose syndrome in North American temperate bats. Using information publicly provided by the US Geological Survey for *Pd* surveillance in a map application (https://whitenosesyndrome.org/where-is-wns), we determined the year of *Pd* introduction within each state of capture as the earliest year of confirmed or suspected *Pd* occurrence. The year of confirmed or suspected *Pd* detection is indicated by county color within the available US Geological Survey map application (https://whitenosesyndrome.org/where-is-wns). Therefore, we used the earliest confirmed or suspected *Pd* detection year (as indicated visually by county color on the map application at https://whitenosesyndrome.org/where-is-wns and confirmed through model predictions through the US Geological Survey [19]) as the year of *Pd* introduction within each state of collated big brown bat capture data. Using the year of *Pd* introduction for each state as the baseline, we subtracted the year of each individual *E. fuscus* capture from this timepoint to standardize the timing of pathogen spread across the eastern USA. Therefore, the year of *Pd* introduction was set at ‘0’, with negative integers representing years prior to *Pd* introduction and positive integers representing years following *Pd* introduction within each state of capture. From this variable (“years_Pd”), we created another variable (“disease_time_steps”) categorizing pathogen occurrence timing into invasion time-steps [3,^12^,20]. These time-steps included pre-invasion years (< 0 years since *Pd* introduction), invasion years (0 – 1 years since *Pd* introduction), epidemic years (2 – 4 years since *Pd* introduction) and established years (5 + years since *Pd* introduction). We used these time-steps to remain consistent with pathogen occurrence time groups within the white-nose syndrome literature [3,12], *in lieu* of unavailable pathogen prevalence data.

To validate data (Fig. 6), we removed individual *E. fuscus* capture entries that had inconsistencies in reporting using the filtering tool in Microsoft Excel. For example, if an *E. fuscus* record was marked as a male with a female reproductive status (e.g., pregnant male), it was eliminated from the dataset. Additionally, if a juvenile (bat within its first summer of life) female bat was marked with an adult-only reproductive status (*i.e.,* pregnant, lactating or post-lactating), the entry was removed. Juvenile males were allowed an adult reproductive status (testes-descended) because they begin to physically present as reproductive in late summer/early fall. These validation steps removed 29 additional entries from the dataset.

We also explored the ranges of mass (g) and forearm lengths (mm) within the dataset. Average adult *E. fuscus* body mass and forearm lengths have been reported near and around 17.6 g [21] and 45.8 mm [22]. Juvenile body mass and forearm length have historically averaged 12 g and 45.2 mm [22]. Within the data collated here, adult body mass and forearm length averaged 18.8 g (range: 9.2 g to 34.5 g) and 46.5 mm (range: 32.7 mm to 59.0 mm) and juvenile mass and forearm length averaged 15.7 g (range: 6.0 g to 29.7 g) and 46.0 mm (range: 32.0 mm to 55.0 mm). Being that *E. fuscus* masses and forearm lengths have not been collated with such a large sample size in the past, we kept all remaining entries within the dataset due their close proximity to historical averages for mass and forearm lengths. Following data validation, 30,496 individual bat captures across 3,567 unique sites remained within the final dataset (Fig. 6) [1].

## Ethics Statements

Collection and collation of the data described here did not involve human subjects, animal experiments, or data collection through social media platforms. All data contracts and distribution policies required by primary data sources were complied.

## CRediT authorship contribution statement

**Molly C. Simonis:** Conceptualization, Methodology, Validation, Formal analysis, Investigation, Data curation, Writing – original draft, Writing – review & editing, Visualization, Supervision, Project administration. **Lynn K. Hartzler:** Conceptualization, Methodology, Supervision, Writing – review & editing. **Joshua Campbell:** Data curation, Writing – review & editing. **Timothy C. Carter:** Data curation, Writing – review & editing. **Lisa Noelle Cooper:** . **Katelin Cross:** Data curation, Writing – review & editing. **Katherine Etchison:** Data curation, Writing – review & editing. **Traci Hemberger:** Data curation, Writing – review & editing. **R. Andrew King:** Data curation, Writing – review & editing. **Richard J. Reynolds:** Data curation, Writing – review & editing. **Yasmeen Samar:** Data curation, Writing – review & editing. **Michael R. Scafini:** Data curation, Writing – review & editing. **Sarah Stankavich:** Data curation, Writing – review & editing. **Gregory G. Turner:** Data curation, Writing – review & editing. **Megan A. Rúa:** Conceptualization, Methodology, Supervision, Visualization, Writing – review & editing.

## Declaration of Competing Interest

The authors declare that they have no known competing financial interests or personal relationships that could have appeared to influence the work reported in this paper.

## Data Availability

Big brown bat (Eptesicus fuscus) capture records before and after white-nose syndrome (Original data) (Dryad). simonimc/Eptesicus_fuscus_big_brown_bat_data_descriptor_figures: Eptesicus_fuscus_big_brown_bat_data_descriptor_figures (Reference data) (Zenodo).
